# Evaluation of palatal donor site wound healing with concentrated growth factor after gingival graft procedures

**DOI:** 10.1186/s12903-025-06898-z

**Published:** 2025-10-21

**Authors:** Resül ÇOLAK, Nihal ÖZÇELİK, Merve KÜÇÜKOĞLU ÇOLAK

**Affiliations:** Department of Periodontics, Faculty of Dentistry, Zonguldak Bülent Ecevit University, Zonguldak, Turkey

**Keywords:** Pain, Palate, CGF, Gingival grafts, Wound healing

## Abstract

**Objectives:**

Healing of the palatal donor site and patient comfort after free gingival grafting are a challenge for clinicians. This retrospective study aimed to evaluate and compare the effects of concentrated growth factor (CGF) and gelatin sponge on palatal wound healing and patient comfort following free gingival graft harvesting.

**Materials and methods:**

Data were collected from 33 patients who underwent palatal wound management with CGF and absorbable gelatin sponge after free gingival graft harvesting. Epithelialization of the wound was assessed using the H₂O₂ test, while pain intensity was measured using the Visual Analog Scale (VAS). Both evaluations were performed on the 3rd postoperative day and at weekly intervals (1st, 2nd, 3rd, and 4th weeks). Analgesic consumption was recorded in three periods: T1 (days 1–3), T2 (days 3–7), and T3 (days 7–14).

**Results:**

VAS pain scores did not differ significantly between the groups at any time point, though control group scores were higher on day 3. All patients had complete epithelialization by week 4, with the test group showing faster epithelialization at weeks 2 and 3 (not statistically significant). The control group required significantly more analgesic tablets than the test group on postoperative day 3 and throughout the healing period.

**Conclusions:**

Palatal wound healing, pain perception, and analgesic consumption were comparable between CGF and absorbable gelatin sponge dressings.

## Introduction

Mucogingival deformities, which are common around teeth and implants, particularly in the form of gingival recession and lack of keratinized mucosa, are commonly treated with autogenous gingival grafts, which are still considered the gold standard for mucogingival surgery [1–4]. The major types of autogenous gingival grafts include free gingival graft (FGG), subepithelial connective tissue graft (SCTG), and de-epithelialized gingival graft (DGG) [5]. Intraoral donor sites for harvesting autogenous gingival grafts are most commonly the hard palate and the maxillary tuberosity [6]. Each donor site has specific advantages and limitations depending on the individual clinical case. Grafts harvested from the maxillary tuberosity are composed of dense collagenous fibers covered by a well-defined keratinized epithelium, which helps reduce postoperative graft shrinkage and allows for better dimensional stability [7, 8]. However, it has also been reported that tuberosity grafts may lead to the formation of unesthetic white tissue patches over time, possibly due to hyperplastic reactions [7, 9]. Moreover, the relatively small size of the tuberosity area, especially in patients with limited mouth opening and/or in the presence of a third molar, can make access difficult and limit the dimensions of the harvested graft [10]. Consequently, in cases where graft thickness is the priority, the tuberosity region is often preferred. In contrast, when longer grafts are required, the palatal area is usually the donor site of choice [11].

After FGG harvesting, the palatal donor site is typically left to heal by secondary intention. However, with various techniques used for connective tissue graft (CTG) harvesting, primary closure of the donor area is achievable [12, 13]. Nevertheless, de-epithelialized gingival grafts, which contain dense and compact connective tissue close to the epithelium, are often preferred because of their favorable histological characteristics [14, 15]. In cases where the palatal mucosa is thin, the overlying flap used for primary closure may also be thin, thereby increasing the risk of flap necrosis [15]. For this reason, techniques that allow secondary healing of the donor site during connective tissue graft harvesting are considered safe and successful alternatives. Since secondary intention healing of the palatal donor site may lead to postoperative complications, appropriate wound management is essential. The healing process of the palatal donor area typically takes about 2 to 4 weeks following graft harvesting [16]. This process involves fibroblast proliferation, collagen synthesis, angiogenesis, and wound contraction, while revascularization, immune response, and epithelial cell proliferation also play critical roles in achieving optimal wound healing [17]. Various hemostatic and regenerative agents have been proposed to minimize complications, reduce patient morbidity, alleviate postoperative pain, and accelerate healing [18, 19]. To specifically reduce prolonged bleeding and pain, both traditional and advanced materials have been investigated, including absorbable synthetic collagen [20], absorbable gelatin sponges [21, 22], cyanoacrylate adhesives [23], and oxidized regenerated cellulose [24]. More recently, the use of platelet concentrates, such as platelet-rich fibrin (PRF) and concentrated growth factors (CGF), has also been explored for their regenerative potential [22, 25].

Platelet concentrates have been one of the biomaterials used in tissue repair since the 1970 s [26, 27]. Over time, preparation protocols have been developed to enhance their biological efficacy, leading to the classification of platelet concentrates into different generations, which has contributed to the emergence of more reliable and effective biomaterials. The first-generation platelet concentrate used in dentistry is platelet-rich plasma (PRP), which is typically prepared using a two-step centrifugation protocol. In this method, platelet activation and fibrin formation are achieved by the addition of bovine thrombin and calcium [28]. However, concerns about the potential adverse effects of these exogenous components on wound healing, as well as the complex and time-consuming preparation process, have necessitated the development of more biocompatible and practical alternatives. In response to these limitations, Choukroun et al. introduced PRF, a second-generation platelet concentrate characterized by the formation of a more stable fibrin clot without the use of exogenous additives [29].

Concentrated Growth Factor, classified as a third-generation platelet concentrate, was developed by Sacco in 2006. It is obtained without the use of any exogenous additives, eliminating the risk of contamination during preparation [29]. The most distinguishing feature of CGF compared to other platelet concentrates is its ability to isolate a larger and denser fibrin matrix rich in growth factors, due to its variable-speed centrifugation protocol [30, 31].

CGF supports soft tissue healing by several mechanisms, due to its content of CD34-positive stem cells [32], activated platelets, leukocytes, and key growth factors such as TGF-β, VEGF, and PDGF [32–34]. During the fractional centrifugation, platelets release growth factors and cytokines that accelerate the formation of granulation tissue. Meanwhile, the stem cells and monocytes present in CGF may migrate to the wound site where they can differentiate into macrophages, thereby facilitating the healing process [30, 35]. With these properties, CGF acts as both a biological barrier and a reservoir of growth factors, promoting the regeneration of both superficial and deep soft tissue wounds.

A recent systematic review highlighted the potential of CGF to support the adhesion, proliferation, migration, and differentiation of various cell types, including periodontal ligament cells, apical papilla stem cells, dental pulp cells, and osteoblasts [33, 36]. This suggests that CGF plays an important role in wound healing and tissue regeneration in the treatment of oral diseases. CGF has found wide clinical applications as a biologically inductive material in the treatment of bone defects, gingival recession, osseointegration, regeneration, and tissue repair [37]. To the best of the author’s knowledge, studies evaluating the effect of CGF on palatal wound healing after FGG or DGG harvesting remain limited. This retrospective study aims to assess the effect of using a CGF membrane as a palatal dressing on the completion of epithelialization at the palatal donor site, in comparison to the use of absorbable gelatin sponge alone. Additionally, as a secondary outcome, the study evaluates patients’ postoperative pain scores and analgesic consumption.

## Materials and methods

This study was designed as a retrospective cohort study comparing postoperative outcomes of patients treated with CGF or an absorbable gelatin sponge (Spongostan^®^) following palatal graft harvesting procedures. Ethical approval for this retrospective study was granted by the Non-Interventional Clinical Research Ethics Committee of Zonguldak Bülent Ecevit University (Decision No. 2024/17 − 16, dated 02 October 2024). Clinical trial number and consent to publish are not applicable. Informed consent was formally obtained from all patients prior to treatment initiation. However, due to the retrospective nature of the study, no further consent was required.

### Sample, groups, and criteria

The study included patients who underwent palatal soft tissue graft harvesting for mucogingival deformity treatment at the Periodontology Department of Zonguldak Bülent Ecevit University between March 2022 and March 2023. The sample size of the study was calculated using the G^*^Power program (version 3.1.9.7, Franz Faul, University of Kiel, Kiel, Germany), based on the study by Lektemur Alpan et al. [38]. Accordingly, with an α error probability of 0.05 (α err prob) and a power of 0.90 (1-β err prob), the actual power of the study was calculated to be 0.9183988 if at least 28 samples (14 per group) were included (non-centrality parameter λ = 3.1220596 and a critical T = 1.70562). To further increase the power of the study, at least two more patient were added to each group, resulting in a total of 33 samples divided into 2 treatment groups based on the material applied: CGF or absorbable gelatin sponge (Spongostan^®^). Only patients who attended regular follow-up visits for one month after surgery were included. During this period, healing of the palatal donor area and patient comfort were evaluated. Inclusion criteria for the study are as follows:

- Harvesting of epithelialized or de-epithelialized free gingival graft.
- Age ≥ 18 years,
- Having healthy systemic condition,
- No smokers,
- Patients who regularly attended postoperative follow-up appointments.
- No use of medications that impair tissue healing and affect pain perception.
- No use of overdentures or partial dentures covering the donor area.

Patients who did not meet at least one of the inclusion criteria were excluded from the study.

### Surgical procedure

Initially, the area with mucogingival deformity was prepared by the planned surgical procedures. Subsequently, the required graft size was measured and transferred to a sterile aluminum foil template. Thereafter, the donor site was anesthetized using 0.5 mL of 4% Articaine HCl with 1: 100,000 epinephrine bitartrate (Maxicaine Fort, VEM, Istanbul, Turkey). Due to the structural characteristics of the palatal tissue, a free gingival graft was harvested from the hard palate, extending from the first premolar to the first molar tooth. The approximately 2-mm-thick graft, consisting of epithelium and a thin layer of underlying connective tissue, was harvested using sharp dissection [21]. Following graft harvesting, the dimensions of the graft (width and length) and the thickness of the residual palatal mucosa at the midpoint of the wound area were measured using a UNC-15 periodontal probe (Hu-Friedy, USA).

In the test group, the exposed donor site was covered with CGF obtained from autologous blood from the patient, while in the control group, an absorbable gelatin sponge (Spongostan^®^) suitable for the wound size was applied. After manual pressure was applied to the wound area, the palate was fixed with pressure sling sutures in both the test and control groups. No additional surgical acrylic stent was provided to the patients. Depending on the surgical requirement, the graft was then applied to the recipient site either as harvested or following extraoral de-epithelialization. All surgical procedures were performed by the same experienced periodontologist (R.Ç), ensuring standardization across cases.

### Preparation of CGF

**Fig. 1:**
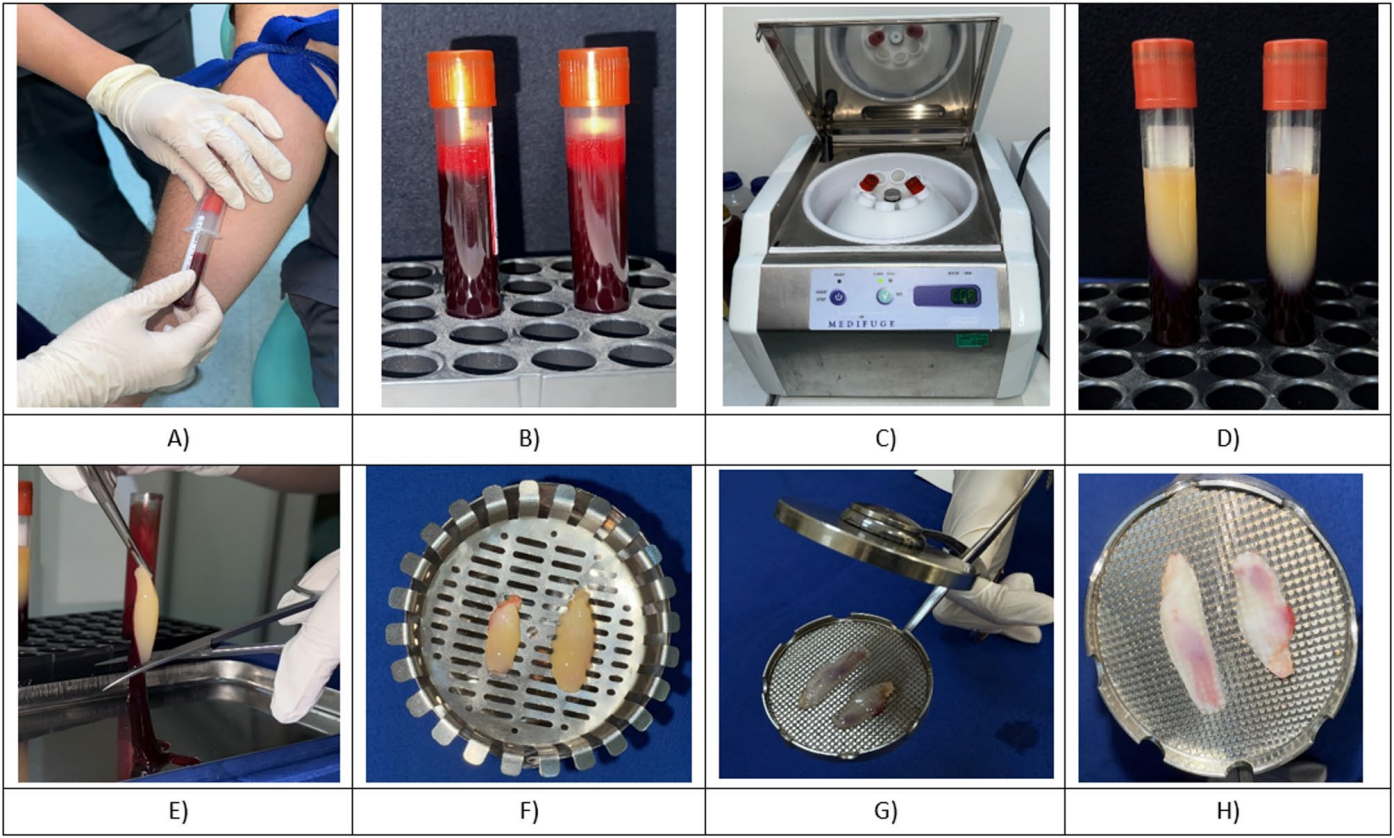
**A** Venous blood collection from the patient, (**B**) Blood samples immediately after collection, before centrifugation, (**C**) Placement of the tubes into a specialized centrifuge (Medifuge) for the CGF protocol, (**D**) Blood after centrifugation, (**E**) Extraction of the CGF clot from the tube using sterile tweezers, (**F**) CGF clots placed in a compression device, (**G**) CGF clots being compressed to form membranes, (**H**) CGF membranes ready for clinical application

Intravenous blood taken before surgery was collected in two 10 mL glass-coated plastic tubes without added anticoagulant. The blood samples were centrifuged (Medifuge, Silfraden, Italy) with 30-s acceleration, 2 min at 2700 rpm, 4 min at 2400 rpm, 4 min at 2700 rpm, 3 min at 3000 rpm, and a 36-s deceleration cycle, as previously described [39]. After centrifugation, the blood in the centrifuge tube was separated into three layers from top to bottom: the platelet-poor plasma layer, the CGF layer, and the red blood cell layer [40]. The CGF clot was obtained by cutting with sterile scissors to include approximately 1.5 mm of the underlying layer, then compressed in a special box and applied to the donor site in a 1 mm layer [15] (see Fig. 1).

### Postoperative care

All patients were advised to use 0.12% chlorhexidine mouthwash twice daily for 1 week. Patients experiencing severe pain were advised to use Parol 500 mg (Atabay, Istanbul, Turkey). In addition, patients were informed both verbally and in writing about avoiding hot and hard foods and postoperative precautions.

### Follow-up protocol

All patients were followed up on the 3rd day, 7th day, 14th day, 21 st day, and 1 month after the surgery. The following parameters were evaluated during the follow-up:

 [1] Epithelialization of the donor area.

 [2] Post-surgical pain levels of the patients.

 [3] Post-surgical analgesic consumption of the patients.

The epithelialization of the donor area was evaluated by applying hydrogen peroxide to the palatal wound with the peroxide test described by Marucha et al. [41] The procedure was performed as follows: after the donor area was gently dried with airflow, the wound was rinsed with 3% H_2_O_2_ with a triple syringe and the operator waited for foaming (bubbles). The absence of epithelialization was characterized by the formation of bubbles after contact of hydrogen peroxide with the wound [42]. The test was performed until complete epithelialization (CE) was achieved. Epithelialization was recorded as a binary variable (yes/no). The outcomes were recorded as non-epithelialized (positive for bubbles) and completely epithelialized (negative for bubbles).

Post-surgical pain levels were determined using a 100 mm visual analog scale (VAS) with endpoints labeled “no pain” and “severe pain”. Participants marked their pain level on the scale, and scores were converted to a 0–10 scale using a ruler [43].

Analgesic consumption (number of tablets) was recorded during three periods: T1 (first 3 days), T2 (between days 3 and 7), and T3 (between days 7 and 14). Bleeding associated with palatal wounds during the first week after surgery was recorded as an early complication.

### Statistical analysis

Statistical analysis was performed using IBM SPSS advanced statistics (Statistical Package for the Social Sciences, version 27, SPSS Inc., Chicago, IL). The data were analyzed for normality using the Shapiro-Wilk test. Pain scores, graft dimensions, remaining palatal tissue thickness, and analgesic consumption data were found to be non-normally distributed. Nonparametric variables were shown as median and range values, and the Man-Whitney U test was used to determine the difference between groups. Freidman’s test was used for within-group evaluation of repeated measurements, and Dunn’s test was used for post hoc pairwise comparisons. Categorical variables were shown as frequency and percentage, and intergroup comparison was performed with the Chi-Square test.

## Result

The data of a total of 33 patients (7 males, 26 females; mean age: 43.2 ± 12 years) were collected from the archive of the Periodontology department. 17 subjects (13 females, 4 males) were suitable for the test group (CGF) and 16 subjects (13 females, 3 males) for the control group (absorbable gelatin sponge). There were no statistically significant differences between the groups in terms of age (*p* = 0.367) and sex distribution (*p* = 0.737).

No significant differences were found between the groups in terms of graft length (*p* = 0.342), graft width (*p* = 0.477) or residual palatal tissue thickness (*p* = 0.76). The measurements of graft length, graft width, and residual palatal tissue thickness are summarized in Table 1 by group.

**Table 1:** Comparison of graft dimensions between control group and test group

GRAFT DIMENSIONS	CONTROL		TEST		
	Median(Min-Max)	IQR	Median(Min-Max)	IQR	*p*
Length	23(10–31)	14.3	15(9–25)	9	0.342
Width	5.5(4–11)	3	6(4–13)	4	0.477
Graft thickness	1.96(1.5–2.6)	0.41	1.85(1-2.85)	1.27	0.285
Residual palatal tissue thickness	2.13(1–4)	0.7	2(1.5–4.8)	0.5	0.756

Mann-Whitney *U* test; comparisons between test and control groups

**Fig. 2:**
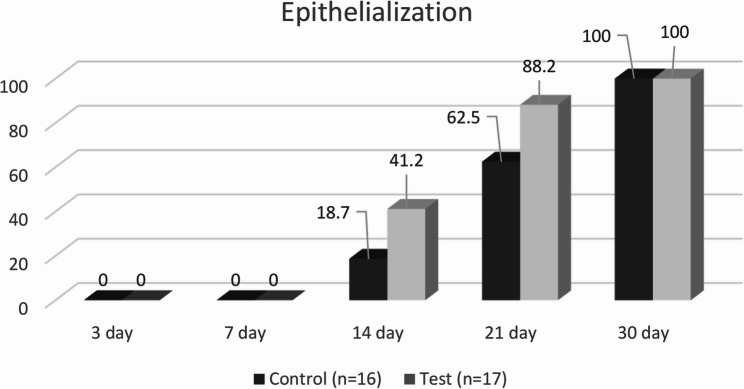
Percentage of epithelialization in control (*n* = 16) and test (*n* = 17) groups at different time points (3, 7, 14, 21, and 30 days)

**Fig. 3:**
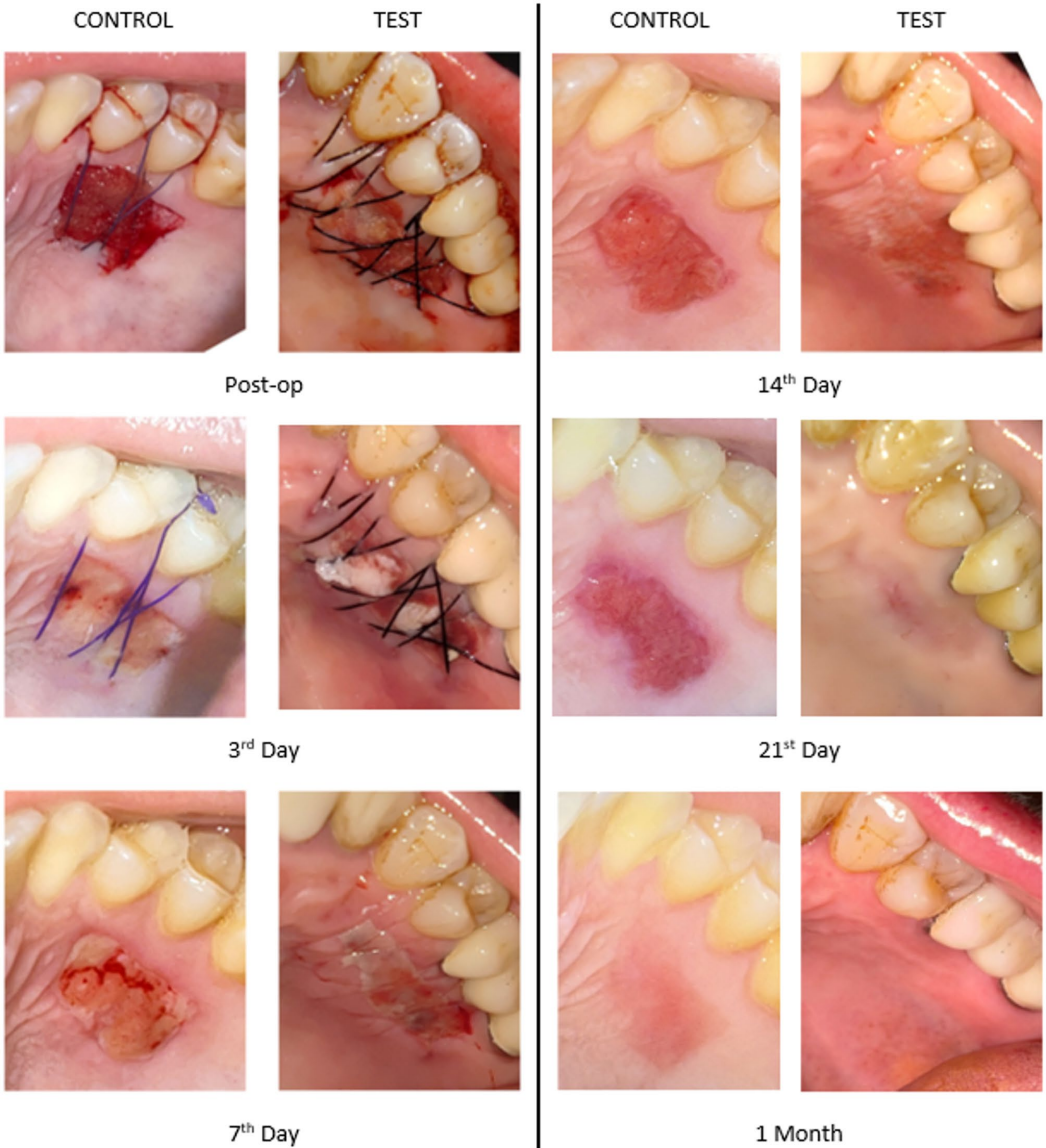
Clinical photographs of palatal donor sites from the Control (Absorbable Gelatin Sponge) and Test (CGF) groups at postoperative 3rd, 7th, 14th, 21 st day and 1 month

The results of epithelialization completion over time in both groups are shown in Table 2. On postoperative days 3 and 7, none of the patients in either group showed complete epithelialization. At day 14, complete epithelialization was observed in 18.7% of the Control Group and 41.2% of the Test Group, with no significant difference between the groups (*p* = 0.259). At day 21, complete epithelialization was present in 62.5% of the Control Group and 88.2% of the Test Group; however, the difference was not statistically significant (*p* = 0.118). In contrast, by the end of the first postoperative month, all patients in both groups had achieved complete epithelialization (see Figs. 2 and 3).

**Table 2:** Comparison of complete epithelialization of the palatal donor site according to groups

	Epithelialization	Control (*n* = 16)	Test (*n* = 17)	
		*N* (%)	*N* (%)	*p*
Post-op 3 days	No	16 (% 100)	17 (%100)	NC
Post-op 7 day	No	16 (%100)	17 (%100)	NC
Post-op 14 day	No	13 (% 81.3)	10 (%58.8)	0.259
	Complete	3 (%18.7)	7 (%41.2)	
Post-op 21 day	No	6 (%37.5)	2 (%11.8)	0.118
	Complete	10 (%62.5)	15 (%88.2)	
Post-op 1 month	Complete	16 (%100)	17 (%100)	NC

^X2^: Chi-square test *NC*: Not Calculated

**Fig. 4:**
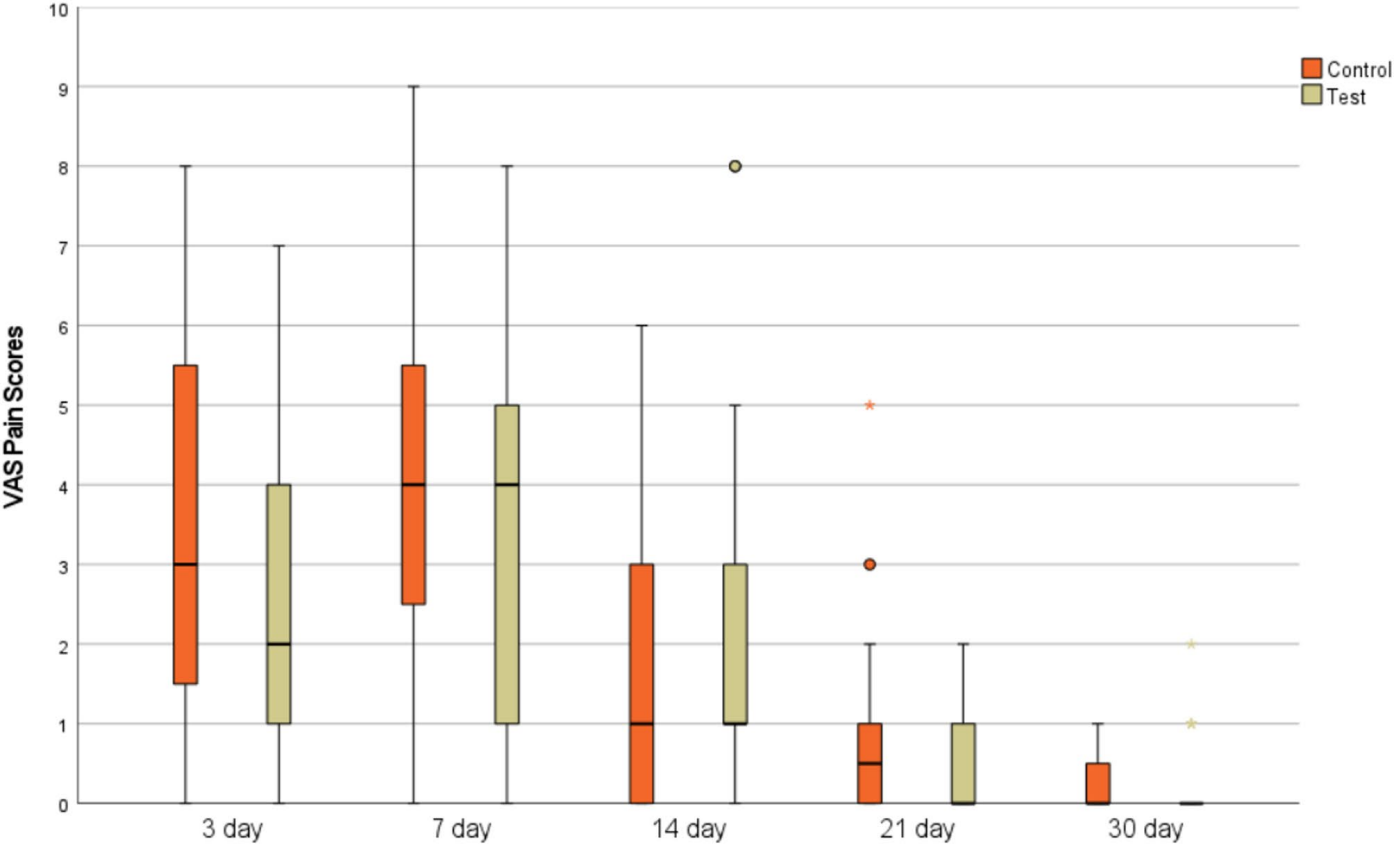
Box-whisker plot of the VAS pain scores changes in control and test groups over time (3, 7, 14, 21, and 30 days)

The statistical analysis results of within and inter-group VAS pain scores at different time points following the surgical procedure for Control and Test groups are presented in Table 3. VAS scores showed a statistically significant decrease over time in Control Group (*p* = 0.001) and Test Group (*p* = 0.001), while no significant change was observed between Control and Test Group at postoperative day 3 (*p* = 0.390), day 7 (*p* = 0.536), day 14 (*p* = 0.460), day 21 (*p* = 0.522), or month 1 (*p* = 1.000) (see Fig. 4).

**Table 3:** VAS pain score statistical analysis results of different time points

	Control			Test			
	Mean ± SD	Median (Min-Max)	95% CI	Mean ± SD	Median (Min-Max)	95% CI	*p*
Post-op 3. day	3.69 ± 2.72	3^a^ (0–8)	2.24–5.14	2.76 ± 2.27	2^a^ (0–7)	1.59–3.94	0.298^t^
Post-op 7. day	4.13 ± 2.55	4^a^ (0–9)	2.76–5.49	3.53 ± 2.67	4^a^ (0–8)	2.16–4.90	0.518^t^
Post-op 14. day	1.69 ± 1.95	1^b^ (0–6)	0.64–2.73	2.35 ± 2.62	1^a^ (0–8)	1.01–3.70	0.448^u^
Post-op 21. day	0.94 ± 1.38	0.5^c^ (0–5)	0.20–1.68	0.53 ± 0.71	0^b^ (0–2)	0.16–0.90	0.509^u^
*p*	0.001^f^			0.001^f^			

^u^: Mann-Whitney *U* test; comparison between groups at the same time point. ^t^: İndependent t test ^f^: Friedman test; intra-group comparison time-dependent

Values with different superscript letters (^a, b, c^) in the same column are significantly different (*p* < 0.05)

**Fig. 5:**
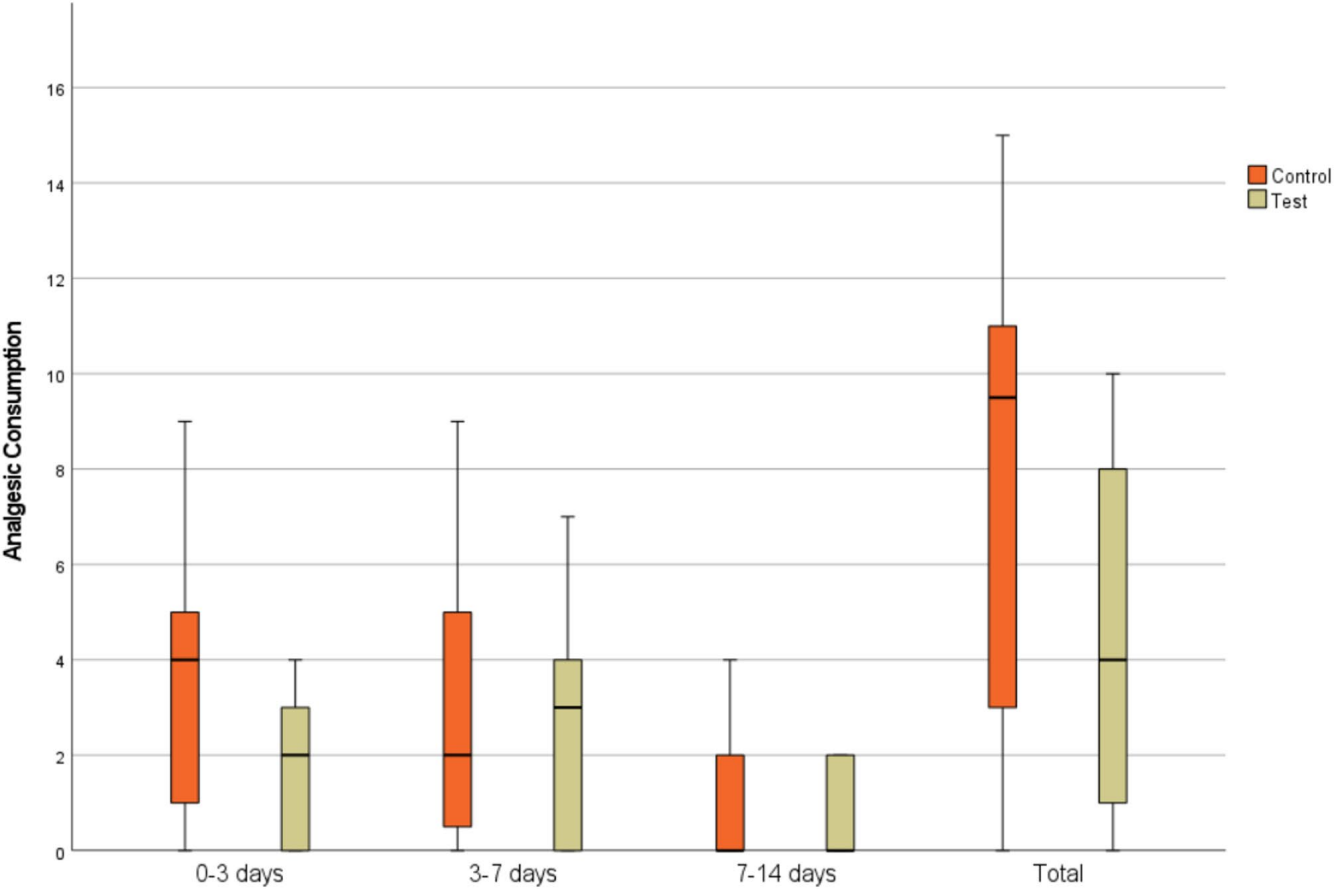
Box-whisker plot of analgesic consumption in control and test groups at different time intervals (3, 3–7, 7–14 days, and total)

The analgesic consumption of patients in both groups was evaluated at specific postoperative intervals, as shown in Table 4 (see Fig. 5). On postoperative day 3, the Control Group showed a significantly higher analgesic consumption, compared to the Test Group (*p* = 0.023). Between postoperative days 3–7, no significant difference in analgesic consumption was observed between the groups (*p* = 0.581). From postoperative days 7–14, the difference in analgesic consumption remained non-significant (*p* = 0.731). In total, the Control Group consumed a significantly higher amount of analgesics compared to the Test Group (*p* = 0.046). Intra-group comparisons revealed a significant time-dependent decrease in analgesic consumption in both groups (*p* < 0.001).

**Table 4:** Evaluation of analgesic consumption within and between groups

	Control			Test			*p* ^u^
	Mean ± SD	Median (Min-Max)	95% CI	Mean ± SD	Median (Min-Max)	95% CI	
Post-op 3 days	3.63 ± 2.63	4^a^ (0–9)	2.22–5.03	1.71 ± 1.57	2^a^ (0–4)	0.89–2.51	0.023
Post-op 3–7 days	3.06 ± 2.99	2^b^ (0–9)	1.46–4.66	2.35 ± 2.29	3^a^ (0–7)	1.18–3.53	0.581
Post-op 7–14 days	0.94 ± 1.28	0^c^ (0–4)	0.25–1.62	0.71 ± 0.92	0^b^ (0–2)	0.23–1.18	0.731
Total	7.56 ± 4.61	9.5 (0–15)	5.10–10	4.76 ± 3.89	4 (0–10)	2.76–6.77	0.046
*p*	0.001^f^			0.001^f^			

^u^: Mann-Whitney *U* test; comparisons between test and control groups. ^f^: Friedman test; Intra-group comparison time-dependent

Values with different superscript letters (^a, b, c^) in the same column are significantly different (*p* < 0.05)

## Discussion

This study evaluated, the effects of concentrated growth factor and absorbable gelatin sponge application on palatal wound epithelialization, postoperative pain perception, and analgesic consumption after free gingival graft harvesting. The results showed no statistically significant differences between the groups for completion of epithelialization and comparison of VAS scores. Despite the lack of statistical significance, patients treated with CGF appeared to experience less postoperative discomfort and demonstrated a more consistent trend toward earlier epithelial coverage. Furthermore, the number of analgesics used by the CGF group through postoperative day 3 and throughout the healing period was significantly lower than that of the control group. Although the initial hypothesis suggested that CGF would result in superior clinical outcomes compared to absorbable gelatin sponge, the absence of statistically significant differences led to failure to reject the null hypothesis. This may be due to factors such as the limited sample size, individual variability in healing responses, and the possibility of a Type II error, which could have reduced the power to detect meaningful differences despite observed clinical trends.

The palatal region is primarily preferred as a donor site because it offers better volume stabilization and offers a wide band of keratinized tissue with ideal thickness [44, 45]. However, despite these anatomical and clinical advantages, palatal graft harvesting is not without limitations. Healing of the palatal donor site typically requires 2 to 4 weeks postoperatively, and complete wound healing is assessed by the extent of re-epithelialization [16, 46].

According to reports in the literature, CGF contains several growth factors, including transforming growth factor-β (TGF-β), vascular endothelial growth factor (VEGF), insulin-like growth factor (IGF), and epidermal growth factor (EGF) [40]. These factors significantly enhance epithelial cell migration and promote the formation of a new vascular network around the wound site [47–49]. In particular, CGF shares many of the same growth factors found in PRF; however, due to its unique fibrin architecture, a dense and elastic three-dimensional network of interwoven fibrous proteins, it offers superior structural integrity and handling properties [50]. Moreover, its fibrin matrix is relatively loose, allowing numerous platelet clusters to reside near the upper region, from which growth factors are gradually released during the slow degradation process, thereby extending their biological activity duration [51]. Studies have also shown that the fibrin meshwork in CGF is enriched with leukocytes, which play a critical role in modulating inflammation and preventing infection [52]. Additionally, the presence of TGF-β, VEGF, and a high concentration of CD34 + cells in CGF has been associated with improved vascular stability [53], neovascularization [54], and immunomodulatory functions [55]. Compared to PRF, CGF not only contains higher concentrations of these growth factors but also provides a longer duration of release, which may offer superior benefits in sustaining tissue regeneration [50]. In addition to accelerating wound healing, platelet concentrates such as PRF and CGF offer significant safety advantages, because they are derived from the patient’s own blood, eliminating the risk of transmissible infections and immune rejection. Previous studies have shown that platelet concentrates can accelerate wound healing by promoting early epithelialization, often within two to three weeks [25, 56]. Feminelle et al. found that 35% of the PRF test group showed complete wound epithelialization by week two, and 100% by week three, with a significant difference compared to the control group [57]. In addition, Aravindaksha et al. reported complete healing at week 3 in 100% of PRF-treated patients, further supporting the positive impact of platelet concentrates on epithelialization [58].

In this study, complete epithelialization was observed in all patients by the end of the first postoperative month. Although no epithelialization was observed in the first 7 days in either group, a higher percentage of patients in the CGF group showed complete epithelialization on days 14 and 21, suggesting a trend toward faster healing compared to the absorbable gelatin sponge group, although this was not statistically significance. In contrast, Yuan et al. (2021) reported a statistically significant improvement in wound epithelialization when CGF was applied to palatal donor sites, compared to an iodoform sponge [49]. Their findings showed that CGF not only accelerated the epithelial healing process, but also contributed to reduced postoperative pain and improved patient comfort [49]. Similarly, our findings demonstrated a greater extent of epithelialization in the CGF group during follow-up visits, aligning with the observations of Yuan et al.; however, the lack of statistical significance may be attributed to differences in study design, such as graft size and other methodological variations.

Postoperative pain and bleeding after graft harvesting are among the most commonly reported adverse effects [16]. According to the literature, various methods have been reported to reduce postoperative discomfort, including palatal stents, collagen-based materials, resorbable sponges, oxidized cellulose, plant extracts, cyanoacrylate, and platelet concentrates such as PRF [21]. Basma et al. demonstrated that the use of a palatal stent was associated with reduced pain levels, lower analgesic consumption, and increased willingness for retreatment during the early postoperative period [59]. However, in the present study, no stent was provided in order to specifically evaluate the isolated effects of CGF and absorbable gelatin sponge on postoperative outcomes. Many authors have preferred to use both the VAS scores and the amount of analgesic consumption to evaluate the intensity of postoperative pain [25, 46, 56]. Similarly, in the present study, both parameters were used to assess patient-reported pain, as the subjective nature of VAS scores underscores the importance of including objective measures such as analgesic intake for a more reliable evaluation of postoperative pain. While no significant difference in VAS scores was observed between the CGF and absorbable gelatin sponge groups, a significant reduction in pain over time was observed in both groups. At each time point, there was no statistically significant difference in VAS scores between the groups. A review of the literature demonstrates variability in postoperative VAS pain outcomes with the use of PRF. While several studies reported significantly lower pain scores in the PRF groups compared to controls [24, 57, 60–62], other studies found no statistically significant differences between groups [17, 22, 63]. Variations in VAS pain scores between studies may be due to differences in study design, control and test group characteristics, and population heterogeneity. Differences in patient characteristics, such as age and gender, are known to influence postoperative pain and morbidity [64]. However, no significant differences in demographic characteristics were observed between the groups in this study. The grafts were used in various root coverage procedures and to treat peri-implant soft tissue deficiencies. In addition, there was no statistically significant difference in graft dimensions (length, width, thickness, and residual palatal tissue thickness) between the control and CGF groups. Since VAS scores reflect patient-reported outcomes, pain perception may have been influenced by variations in surgical context and individual psychological factors such as preoperative anxiety or expectations of postoperative pain. Beaudette et al. [65] also reported that anticipated pain was strongly associated with actual pain experience, with patients expecting more pain tending to report higher VAS scores.

In this study, both the analgesic consumption during the first three postoperative days and the total analgesic consumption were significantly lower in the CGF group compared to the absorbable gelatin sponge group. Similarly, Femminella et al. reported a significant reduction in analgesic consumption over time in patients treated with PRF [57]. While Tavelli et al. [66] found that the use of a collagen hemostatic sponge resulted in less analgesic consumption compared to allowing the site to heal without a dressing, our findings suggest that biologically active materials such as CGF may offer further advantages in minimizing postoperative discomfort and reducing analgesic consumption. CGF contains a high concentration of growth factors such as TGF-β and VEGF, which can accelerate tissue regeneration and reduce local inflammation, thereby alleviating pain. In addition, the dense yet elastic fibrin matrix of CGF might provide a mechanical barrier that protects exposed nerve endings and minimizes irritation at the donor site during the early healing phase. These findings collectively support the potential of CGF as a biologically active dressing material that not only enhances healing but also improves patient-reported comfort in the immediate postoperative period.

Marques et al. (2022) evaluated changes in tissue thickness and volume at palatal donor sites following graft harvesting using a three-dimensional digital method, demonstrating the effectiveness of this non-invasive technique for monitoring the healing process [67]. Similarly, another study reported that superimposing preoperative tomography slices with three-dimensional scanner data provides a reliable assessment of palatal soft tissue [68]. In contrast to these approaches, the methodology employed in our study limited the ability to monitor certain parameters such as tissue contraction, loss, or gain during wound healing. Additionally, this study had certain limitations that should be acknowledged. The H₂O₂ test used to assess re-epithelialization only provides binary information regarding the presence or absence of epithelial coverage, making it impossible to monitor the dynamic progression or rate of re-epithelialization at each time point. Ideally, serial photographic documentation of the wound area during healing would allow for more detailed evaluation of epithelial maturation and surface changes. However, in the present study, incomplete digital documentation and the absence of standardized photographic protocols during the postoperative follow-up period hindered this approach. This represents the main limitation of our investigation.

Furthermore, to the best of our knowledge, there is currently only one published study specifically evaluating the effects of CGF on palatal wound healing, which is not available in English. The limited existing literature demonstrates the necessity of future studies utilizing standardized protocols calibrated wound assessment tools, larger sample sizes, randomized controlled trials, and advanced digital measurement techniques combined with photographic documentation to provide a more comprehensive understanding of CGF’s role in wound healing.

## Conclusions

Within the limitations of the study, it was concluded that.

(i) No significant difference was observed between the CGF and gelatin sponge groups in terms of epithelialization.
(ii) No significant difference was observed between the CGF and gelatin sponge groups in terms of VAS pain scores.
(iii) The application of CGF reduces the consumption of analgesics by patients.

## Data Availability

The datasets used and/or analyzed during the current study are available from the corresponding author on reasonable request.

## Abbreviations

CGF: Concentrated Growth Factor
FGG: Free Gingival Graft
DGG: De-Epithelialized Gingival Graft
SCTG: Subepithelial Connective Tissue Graft
PRP: Platelet-Rich Plasma
PRF: Platelet-Rich Fibrin
CTG: Connective Tissue Graft
VAS: Visual Analog Scale
H₂O₂: Hydrogen Peroxide
CE: Complete Epithelialization
TGF-β: Transforming Growth Factor-Β
VEGF: Vascular Endothelial Growth Factor
IGF: Insulin-Like Growth Factor
EGF: Epidermal Growth Factor
